# Circadian Control of Antibacterial Immunity: Findings from Animal Models

**DOI:** 10.3389/fcimb.2016.00054

**Published:** 2016-05-10

**Authors:** Landry L. Tsoumtsa, Cedric Torre, Eric Ghigo

**Affiliations:** Centre National de la Recherche Scientifique UMR 7278, IRD198, Institut National de la Santé et de la Recherche Médicale U1095, Institut Hospitalier Universitaire Méditerranée-Infection, Aix-Marseille UniversitéMarseille, France

**Keywords:** antibacterial, bacteria, circadian, drosophila, mice, chrono-immunology

## Abstract

Most of the biological functions, including the immune system, are linked to circadian rhythms in living organisms. Changes occurring to biological parameters as the result of these circadian rhythms can therefore affect the outcome of a disease. For decades, model organisms have proven to be a great tool to understanding biological mechanisms such as circadian cycle and immunity. In this review, we created an inventory of the use of model organisms in order to decipher the relation between circadian rhythms and antibacterial immunity.

## Introduction

The use of animals as models is justified for several reasons, including physiological similarity and scientific evidence of biological mechanism conservation between species. Animals share the same environmental conditions as humans and are exposed to environmental pathogens which are similar to those responsible for human diseases. Animals often serve, therefore, as models in immunology in order to investigate pathogenesis relating to bacterial infection. In addition, they are also used to explore host defense mechanisms in response to infection (Conti et al., 2014). Animals are also subject to transitions between day and night, and their immune systems are shown to adapt to these transitions. For example, 8% of macrophage transcripts are produced in a circadian manner (Keller et al., 2009), Th17 cell differentiation in mice is under circadian control via nuclear receptor RORγτ transcription (Yu et al., 2013), and the secretion of TNFα and IL-6 peak in mouse macrophages during the resting phase (Keller et al., 2009). This rhythmic expression of immune components reflects the daily differences between the immune systems of different living organisms.

In this mini-review we aim to review the findings from animal models, with a particular focus on mice and drosophila, which provide links between the antibacterial immune response and circadian rhythms. In addition, we also discuss the contribution of emerging animal models in the study of the relationship between the circadian cycle and anti-bacterial response.

## Molecular basis of the circadian rhythm

Daily oscillations of physiological parameters such as blood pressure, body temperature and hormone secretion levels highlight the existence of circadian rhythms (Lamont et al., 2007; Zhang et al., 2014). These rhythms are found in most living organisms, from cyanobacteria to humans. They allow organisms to anticipate and adapt to nycthemeral variations imposed by the rotation of the earth (Merbitz-Zahradnik and Wolf, 2015). Circadian rhythm periods are approximately 24 h long. They are generated by internal biological oscillators. The main oscillator, termed the central oscillator, is the suprachiasmatic nucleus (SCN), which is located at the base of the hypothalamus. Peripheral oscillators are present in many cells, tissues and organs (Dardente and Cermakian, 2005). The role of the SCN is to coordinate and synchronize different oscillators (Ko and Takahashi, 2006). Genetic screening and the study of mutants have identified a set of genes called the “clock genes,” which are the molecular basis of rhythm generation. Clock genes interact by forming post-transcriptional and post-translational regulatory loops, creating a 24-h oscillation. In the main loop, the *Clock*/*Bmal-1* heterodimer, which constitutes positive limb, promotes *Per* and *Cry* transcription during the daytime. PER and CRY proteins are synthesized in the cytoplasm, dimerize and are translocated into the nucleus where they accumulate. In time, this accumulation will repress their own transcription by negative feedback on the *Clock*/*Bmal-1* complex (Ko and Takahashi, 2006). Post-translational modifications, such as phosphorylation or degradation of PER by CSKNIε/Δ, can also regulate the accumulation of the PER/CRY complex in the nucleus (Curtis et al., 2014). In some organisms, such as Drosophila, TIM binds to the PER/CRY complex and is translocated into the nucleus (Hastings and Herzog, 2004). Other regulatory loops have also been described, including one that involves the nuclear receptors *Rev-erb* α and *Ror* α, which compete to bind to ROREs (Ror Response Elements). *Rev-erb* α binding downregulates *Bmal-1* expression while *Ror* α binding upregulates *Bmal-1* expression (Scheiermann et al., 2013). Although the clock genes can vary between species, the molecular organization, structure and function of these genes are highly conserved (Paranjpe and Sharma, 2005).

In mice and Drosophila, the two most commonly used model organisms, the molecular architecture of these feedback loops shows the presence of a β-Helix Loop Helix domain on *Bmal-1* and *Clock* transcripts. This enables binding on D and E-box motifs of both the clock genes and a set of genes involved in other biological processes. Therefore, binding to these motifs induces their rhythmic expression and directly drives these biological processes. These genes are known as “Clock Control Genes” (CCG) (Logan and Sarkar, 2012; Wang et al., 2013). Among the identified CCG are several immune-related genes including *Stat-3, Stat-5, Erg-1, Nf-kB*, and *Tlr-9* (Logan and Sarkar, 2012; Silver et al., 2012). Therefore, fluctuations in clocks genes expression lead to variation in the expression of these immune-related genes, which in turn may impact the capacity of the immune system. However, mediation by transcriptional promoters is not the only form of communication between circadian rhythms and the immune response. In murine models, it has been demonstrated that CLOCK forms a complex with the p65 subunit of NF-κB. *Clock* overexpression increases phosphorylation, and acetylation of the p65 subunit leads to an enhanced transcriptional activity of *Nf-kb* (Spengler et al., 2012). Furthermore, circadian secretion of melatonin, a clock-controlled humoral factor, may function to modulate the expression of immune-related genes (Deng et al., 2006).

## Evidence of daily variation in the immune response during bacterial infection

Circadian variation in the ability to combat bacteria was highlighted many years ago. In 1969, researchers proved that, depending on when the infection occurs, circadian fluctuations in the mammalian immune system over the course of the day can lead to changes in the host's response. In this study, mice were subcutaneously inoculated with *Diplococcus pneumoniae* at different times: 4 a.m., 8 a.m., 12 noon and 8 p.m. (real time). Regardless of the dose of bacteria administered, the mice infected at 4 a.m. showed higher survival rates than the mice infected at 8 a.m., 12 noon and 8 p.m. (Feigin et al., 1969). A few years later, Wongwiwat and colleagues inoculated mice by intraperitoneally with two strains of pneumococcus (type I, A5 strain and non-encapsulated R36NC strains) and observed that, for mice infected at 4 a.m., it took longer to reach an intense level of bacteremia (10^8^ bacteria/ml) than the time needed for mice infected at 4 p.m. This led to a higher mortality rate in the mice infected at 4 p.m. compared to those infected at 4 a.m. (Wongwiwat et al., 1972). Both studies clearly showed that in mice, antibacterial response levels can differ according to the time of the challenge. This is likely to be due to the presence of a rhythmic phenomenon in one or more biological process involved in the antibacterial response. Possible mechanisms include the daily difference in hormone and/or enzyme secretion by host during infection. Although Wongwiwat and colleagues failed to identify the specific mechanisms underlying the periodicity of the antibacterial response, they nevertheless demonstrated that an intact adrenal gland is needed for a rhythmic resistance to be observed in mouse survival subsequent to pneumococcal infection, since adrenalectomized mice did not display this rhythmic resistance. As adrenal cortex function displays a circadian rhythm, this proves a direct link between circadian rhythm and resistance to bacteria through adrenal cortex hormones. Recently, a histological analysis of the caeca from mice infected by oral gavage with *Salmonella Typhimurium* demonstrated that mice infected during the resting phase presented more severe signs of inflammation than mice infected during the active phase. Inflammatory signs such as submucosal edema, surface erosion, cryptitis, inflammatory exudates and mononuclear infiltrate observed in the caeca of infected mice were associated with an increased expression of the TNFα (pro-inflammatory cytokine), CXCL-1 (neutrophil chemoattractant chemokine ligand 1), LCN-2 (antimicrobial peptide lipocalin-2) genes, the expression levels of which were also shown to be dependent upon the time of infection. Additionally, in keeping with the inflammatory nature of tissues, the caeca from mice infected at 10 p.m. (night, early active phase) were significantly less colonized by *S. Typhimurium* at both 72 and 78 h post-infection than the caeca from mice infected at 10 a.m. (day, early rest phase) (Bellet et al., 2013). Therefore, these results confirm the existence in mammals of a correlation between the time of infection and host response.

**Figure 1:**
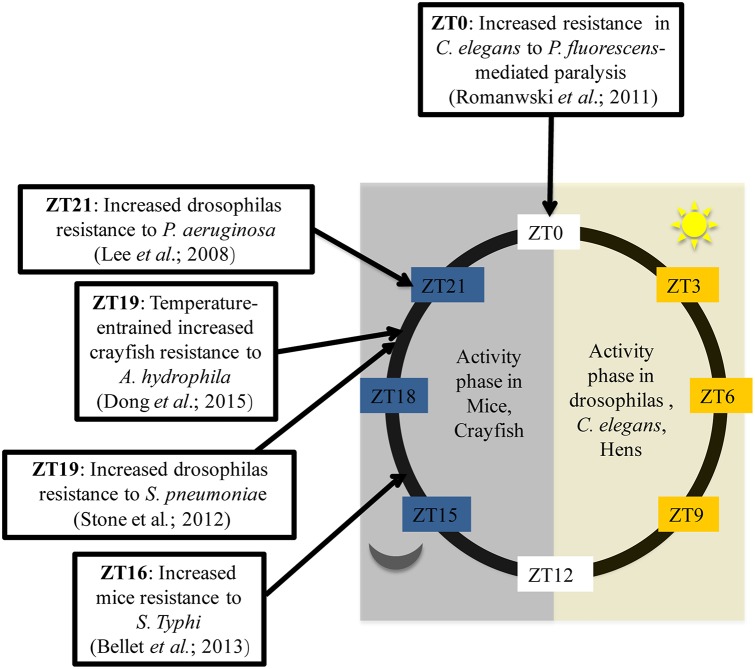
**Time of day higher tolerance to bacterial infection in mice, Drosophilas, *C. elegans*, and crayfish**. ZT is corresponding to an experimental time referring to the onset of a zeitgeber (light or temperature). ZT0 is the transition time from dark to light while ZT12 is the transition time from light to dark. Activity phase is corresponding to the phase where movements or displacements are most recorded.

In addition, in order to thoroughly investigate the circadian variation in the ability to combat bacteria, several studies have been conducted using *Drosophila melanogaster* as a model. Flies kept in 12:12 light-dark conditions (LD flies) and inoculated with *Streptococcus pneumoniae* during the day at ZT7 (Zeitgeber Time (ZT); ZT0 corresponding to lights ON and ZT12 corresponding to lights OFF), die faster than flies infected during the night at ZT19 (Stone et al., 2012). Thus, flies are more resistant to bacterial infection when infected overnight. Similarly, 10 h after inoculation, DD flies (kept in constant darkness) infected at CT5 (Circadian Time 5) exhibited a higher bacterial load with a poor survival rates than flies infected at CT17 (Lee and Edery, 2008). How can this difference been explained? To answer this question, the authors analyzed the post-infection expression of antimicrobial peptide (AMP) genes such as attacin A, defensine, diptericin, drosocin, drosomycin and genes from Pathogen Recognition Receptor (PRR) signaling pathways such as *Pgrp-SA, Pgrp-LC, Pgrp-LB*, and *imd.* The results showed that only the *Pgrp-sa* and *drc* expression patterns differed based upon time of infection. The authors therefore concluded that, based on the time of infection, the circadian rhythm selectively regulates the activation of a limited number of innate immune-related genes which are most likely associated with the production of anti-microbial peptides, found to correlate with bacterial growth kinetics and fly survival rates (Lee and Edery, 2008). Overall, these data reflect the ability of the immune systems of living organisms to adapt to circadian cues. There is a temporal resistance against bacteria which can be explained by circadian modulation on the expression of some immune genes. In mice, the immune system appears to work as if immune alertness fades during the resting phase (day-time in mice), when the animals are less likely to encounter pathogens (Scheiermann et al., 2013) but, as shown by Figure 1, this statement doesn't fit with all living beings. Hence, there is a need to choose the correct model for experiments based on expected outcomes.

## The effect of clock genes on modulating the immune response to bacteria

The development of genetic engineering tools (RNA interference, Knock-Out) has led to the increased use of animal models such as mice and drosophila in chrono-immunology, because they provide an opportunity to directly observe the effects of depleting certain circadian factors, such as clock genes, during the bacterial infection (Table 1). In the following reported experiments, majors clock genes were experimentally knocked-out in order to mimic desynchronized systems. Both wild type (WT) *D. melanogaster* infected at CT17 and *Clock* (−/−) *D. melanogaster* were shown to be resistant to *P. aeruginosa* infection. However, WT *D. melanogaster* infected at CT17 were more resistant to *P. aeruginosa* infection than WT *D. melanogaster* infected at CT5 (Lee and Edery, 2008). Likewise, 72 h after infection with *S. Typhimurium, Clock* (−/−) mice exhibited a bacterial load similar to that found in WT mice infected overnight, but lower than that in WT mice infected during the day (Bellet et al., 2013). Therefore, the absence of *Clock* alters the previously described daily variation of susceptibility to bacteria depending on the time of infection, and allows the immune system to be on high alert against pathogens throughout the day. Furthermore, during infection, *Clock* (−/−) mice produce fewer pro-inflammatory cytokines (TNFα, IFNβ, IL-6, IL-1β) than WT mice (Bellet et al., 2013). In contrast, *Cry* (−/−) mice produce more cytokines (IL-6, TNFα) than WT mice (Narasimamurthy et al., 2012). Thus, pro-inflammatory cytokines secretion is controlled via the expression of clock genes, suggesting that the circadian rhythm can modulate antibacterial response. In another study using mice as models, regardless of the time at which derived *Escherichia coli (E. coli*) LPS was injected, deletion of the *Clock* partner, *Bmal-1*, decreased the survival rate of mice following LPS endotoxic shock (Curtis et al., 2015). The same results were obtained after intraperitoneal infection of *Bmal-1* (−/−) mice with *Listeria monocytogenes*. Indeed, compared to the control, *Bmal-1* (−/−) mice exhibited a decrease on the survival rate during *L. monocytogenes* infection, explained by the presence in mice of an *Bmal-1* dependant rhythmic mobilization of Ly6C^hi^ monocytes (Nguyen et al., 2013). *Per-2* (−/−) mice present significantly increased resistance to *E. coli* LPS endotoxic shock, as 50 h after LPS administration, all *Per-2* (−/−) mice survived the endotoxic shock, while the WT mice died (Liu et al., 2006). Indeed, *Per-2* plays an immune-regulatory role in the production of pro-inflammatory cytokines such as INFγ and IL-1 but not in the production of others cytokines (IL-6, IL-10, TNFα) (Arjona and Sarkar, 2006; Liu et al., 2006). The decrease in INFγ production may be due to the low number of NK cells or the defective NKT function observed in *Per-2* (−/−) mice. Thus, *Per-2* appears to offer circadian control in the innate immune response by acting as a regulator of the production and function of NK cells. In *D. melanogaster*, deletion of *Per-2* renders flies more sensitive to *S. pneumoniae, P. aeruginosa* and *Listeria monocytogenes*. Ten hours after infection, the bacterial load determined by CFU (Colony Forming Units) counts was higher in *Per*-2 (−/−) than in *Clock* (−/−) mice (Shirasu-Hiza et al., 2007; Lee and Edery, 2008). The aforementioned studies appear to give a contradictory role to *Per-2* in the antibacterial immune response in mice and in Drosophila. In mice, however, the challenge was performed using the Gram-negative bacteria ligand LPS, which is certainly immunogenic but is endotoxic and may induce a less complete immune response than the entire bacteria. It would, therefore, be of great interest to conduct further experiments to explore the response of *Per-2* (−/−) mice to entire bacteria.

**Table 1:** **Clock genes mutations consequences for antibacterial immunity**.

**Organisms**	**Genes knock down**	**Effects on immunity**	**Bacterial challenger**	**References**
Drosophila	*Clock*	Increases resistance	*P. aeruginosa*	Lee and Edery, 2008
	*Per-2*	Increases susceptibility	*S.pneumoniae, P. aeruginosa L. monocytogenes*	Shirasu-Hiza et al., 2007; Lee and Edery, 2008
	*Tim*	Pathogen-specific	Increase susceptibility to *S. pneumonia S. marcescens*	Shirasu-Hiza et al., 2007
			Enhanced resistance to *P. aeruginosa*	Lee and Edery, 2008
Mice	*Clock*	Induces less Pro-inflammatory cytokines production	*S. typhimurium*	Bellet et al., 2013
	*Per-2*	Increases resistance to LPS endotoxin shock‘	LPS	Liu et al., 2006
	*Bmal-1*	Increases susceptibility to LPS endotoxin shock	LPS	Curtis et al., 2015
		Increases susceptibility	*L. monocytogenes*	Nguyen et al., 2013
	*Cry*	Upregulates pro-inflammatory cytokines genes expression	LPS	Narasimamurthy et al., 2012

In regards to *Tim*, Lee and colleagues found a survival advantage in *Tim* (−/−) flies infected with *S. pneumonia*, while Shirasu-Hiza described increased susceptibility of *Tim* (−/−) flies to *P. aeruginosa* infection compared to WT flies (Shirasu-Hiza et al., 2007; Lee and Edery, 2008). Interestingly, it appears that *Tim* involvement in the immune response to bacteria could be pathogen-dependent. Indeed, researchers infected *Tim* (−/−) *D. melanogaster* by injecting four different strains of bacteria. Two days after infection with *S. pneumoniae* and *Serratia marcescens, Tim* (−/−) contained more bacteria than WT *D. melanogaster*, while 2 days after infection with *Burkholderia cepacia* and *S. typhimurium, Tim* (−/−)s contained the same levels of bacteria as WT (Stone et al., 2012). The pathogen-dependent actions of *Tim* may be linked to specific defense mechanisms which the flies engage in the presence of a specific type of bacteria. Recently, *Tim* has been shown to regulate the phagocytosis of bacteria in Drosophila but TIM is degraded by light. This explains the increased susceptibility to bacteria observed during the day and the significant phagocytic activity against bacteria observed during the night in Drosophila. Indeed, *Tim* (−/−) *D. melanogaster* have significantly lower phagocytic activity than WT flies after *Staphylococcus aureus* infection, but no difference is observed during *E. coli* infection (Stone et al., 2012). Therefore, *Tim* regulates phagocytosis in a pathogen-specific manner. In their results, while phagocytosis is shown to be regulated by *Tim* expression, melanization and antimicrobial peptide gene expression are not shown to be controlled either by *Tim* expression or by the circadian clock machinery (Stone et al., 2012).

Key clock gene inhibitions always have immediate consequences on the antibacterial response, revealing a circadian control of the host's response to bacterial infection. However, mechanisms and factors allowing for this circadian control are not yet fully understood, particularly because only a few clock genes have already been investigated despite the fact that the circadian machinery in mammals involves more than a dozen genes. Of particular interest is *Rev Erb*α and *Ror*γ*t*, which have been shown through *in vitro* studies on mice cells to have a modulatory effect on the inflammatory function of macrophages and a regulatory role on Th17 lymphocyte differentiation, respectively (Yu et al., 2013; Sato et al., 2014). Thus, additional study is required to fully determine the actors, effectors and mechanisms responsible for the connection between the circadian system and the host's response to bacteria.

## Influence of L/D entrained rhythm on bacterial challenge

Circadian rhythms can be influenced by environmental factors known as zeitgebers. The most important zeitgeber is light. Light cues give the internal clock of the organism information required to reset its phase (El Allali et al., 2013). Light/dark cycling keeps this rhythm synchronized with the environment. The majority of the experiments mentioned above were carried out using animals kept in a 12:12 light/dark cycle. This condition appears to be the most representative of the natural light cycle which living things, including human beings, are subject to in real life conditions. On the other hand, maintaining model organisms under constant conditions (light or dark) may induce desynchronization, with dramatic modifications to the circadian rhythm period, amplitude and/or acrophase. This “free run” of the rhythm can be found in humans with clinical conditions such as cancer, metabolic and autoimmune diseases (Lamont et al., 2007). For example, patients in intensive care units suffer from a disruption to the circadian rhythm often caused by the lack of natural light, noise and medication. This disruption is marked by sleep disturbances and is significantly associated with the occurrence of severe sepsis (Brainard et al., 2015). Furthermore, the immune machinery regulated by circadian rhythms is perfectly synchronized with environmental cues. This regulation may be controlled by melatonin secretion during the dark period. Immune cells contain melatonin receptors and binding melatonin to these receptors promotes the expression of interleukins and IFNγ (Berger, 2008). Likewise, the absence of light stimulates the expression of IFNγ, which is also mediated by melatonin secretion. As demonstrated by Lundkvist in 1999, IFNγ secretion is greater in the suprachiasmatic nuclei of DD rats compared to LD rats (Lundkvist et al., 1999). Lundkvist also observed a loss of rhythmic IFNγ secretion in the CNS of DD rats. In addition, in the absence of melatonin secretion due to constant light exposure, the phagocytic activity of LL rat neutrophils in response to *Escherichia coli* remains rhythmic but is significantly lower than in LD rats. In LD rats, the phagocytic activity of neutrophils increased during the dark period (Hriscu et al., 2002–2003). In Drosophila maintained in constant darkness, fly survival rate depends on the time of infection, which varies in a circadian manner. However, regardless of the time of infection, DD *Tim, Cyc* and *Clock* (−/−) flies survive significantly less often upon infection with *P. aeruginosa* than LD *Tim, Cyc* and *Clock* (−/−) flies. However, DD *Per* (−/−) flies survive better than LD *Per* (−/−) flies (Lee and Edery, 2008). In conclusion, synchronized organisms respond more efficiently to bacterial infection than those which are desynchronized.

## Contribution of emerging animal models

Major clock genes are highly conserved in living beings. Indeed, *Clock, Per* and *Tim* homologs are present in *C. elgans*, crayfish and chickens. (Escamilla-Chimal et al., 2010; Temmerman et al., 2011; Zhang et al., 2016). These alternative models thus became increasingly used to study the link between circadian rhythms and antibacterial immunity. In *Caenorhabditis elegans*, fast paralytic killing caused by *Pseudomonas aeruginosa* or *Pseudomonas fluorescens* occurred more quickly when worms were infected during the night (Romanowski et al., 2011). Less conventional models have also been used to analyze this phenomenon. Temperature affects circadian variations in the immune status of crayfish. Indeed, when placed on a temperature cycle (between 18°C and 24°C), crayfish infected with *Aeromonas hydrophila* at CT19 presented a significantly lower bacterial load 12 h after infection compared with crayfish infected at CT5. This gave the crayfish infected at CT19 a survival advantage (Dong et al., 2015). Furthermore, a microarray analysis of gene expression in the caecum from resistant (3.50 log CFU) or sensitive (1.39 log CFU) chickens to *Campylobacter jejuni* found that *Clock* expression was significantly associated with resistance to *C. jejuni* (Li et al., 2010). Taken as a whole, these studies demonstrate that circadian rhythms play an important role in the host's response to bacterial infections Figure 1. The use of models which are resistant to bacterial infection may provide additional information. Recently, it has been shown that the immortal flatworms, planarians, are resistant to a large number of pathogenic microorganisms (Abnave et al., 2014). Indeed, planarians are able to eliminate 18 different strains of pathogenic bacteria (Abnave et al., 2014). Two species of planarians have been studied: *Schmidtea mediterranea* and *Dugesia japonica*. One mechanism explaining planarians' resistance to bacteria involves *Morn-2*. This resistance mechanism has been proven to be transferable to humans. *Morn-2* forced expression in human macrophages reduced by approximately 70% *Mycobacterium tuberculosis* proliferation in macrophages (Abnave et al., 2014). However, the role of or link between circadian cycle and bacterial resistance in planarians has not yet been investigated. Furthermore, planarians kept in 12:12 light/dark conditions exhibit diurnal variation in the secretion levels of melatonin and serotonin (Itoh et al., 1999; Itoh and Igarashi, 2000). There is also substantial evidence of the existence of an internal clock in planarians which controls many physiological processes, such as fission during asexual reproduction (Sheǐman et al., 2003). This suggests the possible conservation of several mammalian clock genes in planarians. Therefore, planarians may be a good model for identifying the influence of the circadian clock on resistance factors to bacterial infections.

## Concluding remarks

Understanding chrono-immunology may allow the potential benefits to be identified which may improve our ability to efficiently combat bacterial infections. Research using animal models clearly establishes a link between the circadian machinery and antibacterial immunity. Clock genes have proven to be involved in the fight against bacterial invasion, both in vertebrate and invertebrate organisms. Studies conducted thus far are restricted to exploring the impact of the down regulation of such genes on antibacterial response and, yet, it would also be of great interest to examine the over-expression effect on antibacterial response. The use of mutated organisms for circadian clock genes will allow us to understand the influence of circadian rhythms on human antibacterial defenses and help identify critical circadian factors needed to improve resistance to bacteria. Moreover, constant or alternating conditions between light and darkness, the mode of infection and specific host factors (diurnal or nocturnal activity) had undoubtedly a significant influence upon the results presented above. Thus, it seems important to note that the interaction between the circadian rhythm and antibacterial immunity relies on complex interconnected communication systems depending on environmental factors, the circadian rhythm itself, the host and the bacteria. All these parameters must, therefore, be taken into account when selecting experimental conditions in order to obtain standardized and comparable experiments and, of course, study of this interaction cannot be restricted to the study of clock genes. Overall, animal models should continue to be used as the primary tool for investigating the molecular mechanisms underlying the interaction between circadian rhythms and antibacterial immune responses.

## Author contributions

LT conceptualization, original draft. CT original draft. EG conceptualization, original draft, supervision, resourrces, funding, review and editing.

### Conflict of interest statement

The authors declare that the research was conducted in the absence of any commercial or financial relationships that could be construed as a potential conflict of interest.

## Abbreviations

Zeitgeber Time (ZT): Corresponding to an experimental time referring to the onset of a zeitgeber (an environmental cue) such as Light-Dark cycle that synchronizes the internal clocks
Circadian Time (CT): Corresponding to an experimental time without any zeitgeber.
